# P03 Pharmacy technician-led virtual ward rounds: optimizing care for patients on protected antimicrobials

**DOI:** 10.1093/jacamr/dlaf118.010

**Published:** 2025-07-14

**Authors:** Shazia Nazir

**Affiliations:** Leeds Teaching Hospitals NHS Trust, Leeds, UK

## Abstract

**Background:**

The UK-AWaRe classification, based on WHO guidelines (2023), categorizes antibiotics into Access, Watch, and Reserve groups. Reserve antibiotics serve as a last resort, used only for life-threatening infections caused by MDR bacteria. Due to their critical nature, patients on these antimicrobials require daily monitoring and specialist guidance. At Leeds Teaching Hospital, a code system was implemented to ensure the appropriate use of protected antimicrobial agents. Authorization is given through codes granted by infection specialists, with the assigned duration recorded in the medical notes and electronic prescribing chart. The duration of a code depends on factors such as whether use is empirical or directed and the source of infection. Before a code expires, an infection specialist must review the antimicrobial to determine ongoing management. If the infection source and organism are known, the assigned code remains valid for the full duration. Prescribing clinicians remain responsible for ensuring that all protected antimicrobials are reviewed daily and that a valid code is in place. Inconsistent reviews have led to overuse and prolonged continuation of protected antimicrobials.

**Objectives:**

As a Specialist Antimicrobial Stewardship Technician, I identified an opportunity to collect data and review how the code system is being utilized. Daily reviews identified cases where codes required updates, which were escalated to the clinical team to ensure enhanced antimicrobial stewardship.

**Methods:**

Patients were identified using an eMeds report, which provided daily antimicrobial prescribing data. The list was filtered to include protected antimicrobials, patients were reviewed daily enabling prompt follow-up with clinical teams for any cases where codes were missing or had expired. Data collection was conducted from May to November 2024, covering all adult and paediatric inpatients. *Inclusion criteria:* WHO Reserve Category protected antimicrobials; protected antimicrobials used outside of guidelines, either empirically or as directed therapy; and adult and paediatric inpatients prescribed IV protected antimicrobials. *Exclusion criteria:* respiratory or ID consultant-supervised TB treatment; cystic fibrosis/bronchiectasis cases; and haematology patients on prophylactic antifungals.

**Results:**

The audit included 256 patients. A total of 57% had valid Micro codes, while 43% lacked valid codes, leading to continued antimicrobial use without specialist oversight. Among protected antimicrobials, carbapenems (meropenem/ertapenem) were most frequently prescribed, primarily for bacteraemia and sepsis infections.

**Conclusions:**

The audit reveals a gap in antimicrobial stewardship, with 43% of patients on protected antimicrobials lacking valid codes. This highlights the need for improved compliance with the code system to ensure appropriate prescribing and reduce the risk of antimicrobial resistance. The findings also indicate that carbapenems (meropenem/ertapenem) are the most frequently prescribed protected antimicrobials, particularly for bacteraemias and sepsis. Given their critical role as last resort agents, it is essential to enhance daily antimicrobial reviews and strengthen collaboration between clinical teams and infection specialists to optimize use. Additionally, pharmacy input is essential in ensuring real-time monitoring of Micro codes, raising awareness, and enhancing adherence to optimize antimicrobial stewardship and improve patient outcomes.

